# A spotlight on maternal health

**DOI:** 10.1016/j.eclinm.2024.102459

**Published:** 2024-01-24

**Authors:** eClinicalMedicine

Included in this issue is our Series on *Maternal Health in the Perinatal Period and Beyond*, jointly published in *eClinicalMedicine* and *The Lancet Global Health* on Dec 6, 2023. Against the backdrop of off-track maternal mortality estimates for 2030 Sustainable Development Goal (SDG) target 3.1, this four-paper Series provides the scientific basis for embracing new perspectives in tackling global maternal health issues. We hope that this Series will trigger a paradigm shift in both opinion and clinical practice in implementing a holistic, human rights-based approach to addressing maternal ill-health and acknowledging the ever-present power imbalances.

Although this Series is focused on maternal health, it is important to acknowledge that maternal health, and indeed women's health at large, encompasses much more than reproductive health. It is well established that, generally, health conditions that affect women more than men garner less funding. Furthermore, focusing on maternal mortality as the most important measure of maternal health risks ignoring other important aspects of women's health and reinforces deleterious stereotypes about a woman's primary role in society being that of reproduction. We need a broader vision for women's health, as *The Lancet* called for in July 2023, and—whilst acknowledging that mortality is the worst possible outcome—need to prioritise preventing the morbidity incurred by mothers during pregnancy, childbirth, and the postnatal period. This begins with more extensive research. For example, into the various medium-term and long-term sequelae mothers experience after childbirth (eg, depression, urinary and anal incontinence, and sexual dysfunction), as covered in paper 3 of the Series—which calls for the postnatal period to be extended past the current 6 weeks post-birth.

This need for specific research is central to the deliberate choice that this Series focused on women and birthing people and not on the infant. Here, the terms women and mothers are used to reflect all populations with the reproductive capacity for pregnancy and birth, including transgender and gender-diverse people, as well as adolescent girls. With the reproductive rights of women being diminished within several societies globally, it is more important than ever that we acknowledge the distinct and valid needs of the mother within the mother–infant dyad and ensure that these needs are not systematically deprioritised within our health systems.

It is also imperative that policy makers and health-care professionals be mindful that a woman's journey through pregnancy, childbirth, and the postnatal period does not exist in a vacuum. Maternal survival and wellbeing are the product of a complex interplay of various factors, with marginalised groups being more susceptible to maternal ill-health and disability and the intergenerational impacts of poor maternal outcomes. In paper 2 of the Series, Jameela Sheikh and colleagues outline the vulnerability concept in maternal health—with a particular focus on low-income and middle-income countries, where the burden of maternal and perinatal mortality and morbidity is known to be disproportionately high. In this context, the vulnerability concept relates to how maternal outcomes are influenced by interlinked and interdependent vulnerabilities that are either magnified or mitigated by the respective absence or presence of adequate support and coping strategies. Health systems and social forces have the power to either promote or hinder good health and wellbeing. As such, the authors identified reparative strategies with the power to address some of the threats and barriers to good maternal health. These include supplementation with iron for anaemia and calcium for pre-eclampsia; community-based educational interventions, financial incentive programmes, and contraception-promoting interventions. A person-centred approach is crucial to identifying key vulnerability attributes and implementing these targeted reparative interventions. We urge health-care professionals and policy makers to use the vulnerability concept to improve maternal health outcomes, and to implement strategies in partnership with women, their families, and their communities. This collaboration is crucial to designing research and policy that are relevant to the actual vulnerabilities and needs of those affected.

In addition to this person-centred approach that recognises individual vulnerabilities and potential reparative interventions, we concur with Meghan A and colleagues, in paper 4 of the Series, that a more holistic approach to maternal health is also urgently needed. The authors propose recommendations on how to apply an intersectionality lens to maternal health, given that responding only to the inequalities in access, use, and health outcomes—which indeed remain persistent—does not go far enough in addressing an individual's ability to obtain dignified, high-quality maternity care in a world where power and privilege (and conversely oppression and exclusion) drive inequities in more than an additive manner. For example, gendered discrimination during maternity care can be compounded by racism, casteism, ageism, ableism, and transphobia. Most worryingly, Bohren and colleagues identified the absence of any intersectionality-informed strategies or interventions in global maternal health that were measured with quantitative approaches. This represents a major knowledge gap and one that we recommend be prioritised within the maternal health space so that all mothers of all backgrounds and circumstances can be best supported.

The goals and recommendations for this Series sit within a larger call-to-action for a holistic approach to maternal health, the need to reflect on intersectionality and the power and privileges at play within medicine itself (including the structures that favour White, cisgender, heterosexual men), and for ensuring that the needs and outcomes of mothers are not deprioritised within the mother–infant dyad. As emphasised in Edward Kwabena Ameyaw and colleagues’ Comment within the Series, ending preventable maternal death and disability is likely to require a reframing of the postpartum period beyond the customary 6 weeks to capture the longer term complications of childbirth, and to ensure the mobilisation and investment of essential research and resources—a change that we fully support. We urge the maternal health community to take on the recommendations of the Series to best serve mothers and have the best opportunity of achieving the SDG 3.1 maternal mortality target. As a first step, urgent action is needed to extend the customary postnatal period beyond 6 weeks.

